# The quantum-mechanical Coulomb propagator in an L^2^ function representation

**DOI:** 10.1038/s41598-021-96925-0

**Published:** 2021-09-23

**Authors:** Rolf Gersbacher, John T. Broad

**Affiliations:** University of Applied Sciences Esslingen, Robert Bosch Str. 1, 73037 Göppingen, Germany

**Keywords:** Atomic and molecular physics, Quantum mechanics

## Abstract

The quantum-mechanical Coulomb propagator is represented in a square-integrable basis of Sturmian functions. Herein, the Stieltjes integral containing the Coulomb spectral function as a weight is evaluated. The Coulomb propagator generally consists of two parts. The sum of the discrete part of the spectrum is extrapolated numerically, while three integration procedures are applied to the continuum part of the oscillating integral: the Gauss–Pollaczek quadrature, the Gauss–Legendre quadrature along the real axis, and a transformation into a contour integral in the complex plane with the subsequent Gauss–Legendre quadrature. Using the contour integral, the Coulomb propagator can be calculated very accurately from an L$^2$ basis. Using the three-term recursion relation of the Pollaczek polynomials, an effective algorithm is herein presented to reduce the number of integrations. Numerical results are presented and discussed for all procedures.

## Introduction

The long-range Coulomb interaction plays a central role in quantum-mechanical scattering processes with charged particles. Ultra-short laser pulses in the range of femto- and attoseconds allow processes to be monitored in atomic-scales time domains; in particular the final state can be monitored after turning off the laser field, such that the movement of the photoelectron in the field of the charged ion is considered. Phenomena such as above treshold ionization, effects due to carrier envelope phase, and multi-photon ionization, in which an atom or molecule is exposed to an intensive laser pulse for a very short time, require theoretical provision of respective methods to describe the time development of a quantum-mechanical system incorporating Coulomb interactions.

A theoretical treatment of atomic and molecular Coulomb scattering processes, based exclusively on square-integrable functions (L$^2$), is presented in J-Matrix theory. This can be interpreted as a square-integrable analog to the time-independent R-Matrix theory, in which the scattering wave function is expanded in an L$^2$ function space. In recent years, the J-Matrix theory has found widespread significance and applications. Two examples are Konovalov and Bray^1,2^, who calculated electron-impact ionization, as well as the extension to relativistic scattering processes by Alhaidari et al.^3^. For an overview of current applications, we direct the reader to Alhaidari et al.^4^.

The basic functions of the J-Matrix scattering method are the Coulomb Sturmian functions, which have been applied to solve *ab initio* the time-dependent Schrödinger equation in the atto- and femtosecond range *via* close-coupling equation systems. Madronero and Piraux^5^ and Hamido et al.^6^ used this approach to calculate the ionization of atomic hydrogen and helium in a femtosecond laser field.

The aforementioned works show that L$^2$ functions can be successfully used to determine the time development of a system under the influence of a Coulomb field.

For this purpose, we consider the L$^2$ representation of the Feynman propagator^7,8^ for a two-particle Coulomb system: the propagator contains all necessary information to determine the time evolution of a particle in a Coulomb field, i.e., its wave function at time $t'$ can be calculated using knowledge of the wave function at time *t*.

The time propagator can be written formally as an integral and a sum over the eigenstates of the stationary Schrödinger equation and the Coulomb spectral function, bearing in mind that the integrand contains the oscillating function $e^{-iE(t' - t)}$.

1 $$\begin{aligned} K^+(\mathbf {r}\,' , t' ; \mathbf {r} , t) \ = \ \theta (t' - t) \left[ \int \limits _0^\infty \mathrm {d}E ~\Psi _E (\mathbf {r}\,') ~\Psi _E^* (\mathbf {r}\,') ~e^{-iE(t' -t)} + \displaystyle \sum _{n} ~\Psi _{E_n} (\mathbf {r}\,') ~\Psi _{E_n}^* (\mathbf {r}\;\!) ~e^{- iE_n(t' -t)}\right] \end{aligned}$$

$\Psi _E$ characterizes an eigenfunction of positive energy, $\Psi _{E_n}$ characterizes a bound state, and $\theta (t'-t)$ is the Theta function taking a value of 1 for $t' > t$; the superior + symbol shows the retardation realized by the Theta function and the superior star symbolizes a complex conjugation.

For the Coulomb propagator, a range of approximations valid in the limit case of $\hslash \rightarrow 0$ ($\hslash$ represents the Planck constant) were developed in the semiclassical limit; representative works on this subject include Gutzwiller^9^, Tomsovic and Heller^10^ and Manning and Ezra^11^. Unlike these semiclassical approaches, the complete quantum-mechanical Coulomb propagator without approximations is considered here.

In a number of papers^12–15^, it has been shown that not only the bound states (with negative energy), but also the continuum states, can be developed using a single function system of the L$^2$ functions. These solutions to the stationary Schrödinger equation in a L$^2$ basis can be used for the L$^2$ representation of the Coulomb propagator.

Although the Coulomb–Greens operator—which is linked to the Coulomb propagator *via* a Fourier transformation—can be applied by default in scattering computations, the square-integrable analog of the Coulomb propagator has not been studied. To the best of our knowledge, there is no literature on dealing with the Coulomb propagator on an L$^2$ basis.

The goal of this paper is to evaluate numerical methods for the accurate calculation of the Coulomb propagator in an L$^2$ basis and to understand its convergence behavior. J-Matrix-based applications in atomic physics, such as electron-atom-scattering and photoionization or nucleon–nucleon scattering and nuclear decay, can benefit from the results presented herein^4^.

For the expansion of the Coulomb propagator, hydrogen-like Sturmian functions^16^ are used; these contain an additional, often freely available, parameter that increases the flexibility of the system, in particular for applications where various angular momentum states are involved. Closer consideration of (1) reveals that integration over the continuous spectrum poses a great challenge, since a strongly oscillating function occurs in the integrand. The situation is more complicated due to semi-infinite integration intervals and the infinite sum over bound states.

In previous literature, various methods and libraries have been proposed to numerically solve such integrals with oscillating functions. However, if the integrand falls sufficiently “quickly”, standard packages from Matlab, IMSL, Maple, and NAG can be used. Many of these packages trace back to Quadpack^17^, applying a Clenshaw–Curtis algorithm with adaptive partitioning, such that the line of partial sums can be extrapolated with the $\epsilon$ algorithm^18^. Evans^19^, Haider and Liu^20^ and Sauter^21^ also conducted partitioning of the integration interval, as well as Sidi^22^ who partitioned according to the zeros of the integrand before calculating the line of partial sums using a convergence accelerator.

Oscillatory integrals often appear in quantum scattering and in wave propagation in classical electrodynamics. There is a great interest and need for techniques that can cope with such integrals. Besides the aforementioned partitioning approach, methods for evaluating such integrals can be divided into three categories: asymptotic expansions of the integrand and Filon- and Levin-type approaches.

In the asymptotic expansion method the integral is approximated by a series either through repeated integration by parts or by employing a formal expansion of the integrand and then evaluating the integrals term by term. In the Filon method the integral $\int dx f\!(x) \; e^{ig(x)}$ is partitioned into N parts and in each part $f\!(x)$ is approximated by a polynomial. The resulting parts can be integrated either analytically or by standard procedures. The Levin method searches for a function *F* such that $\frac{d}{dx} [ F\!(x) \; e^{ig(x)}] = f\!(x)\; e^{ig(x)}$. This leads to $\int dx f\!(x) \; e^{ig(x)} = \int dx \frac{d}{dx} \; [ F\!(x) \; e^{ig(x)}]$, which can be evaluated trivially. The task to work out the integral is shifted to solve a differential equation for the unknown function F.

A survey of these methods can be found in Deaño, Huybrech and Iserles^23^, where further work on the topic is also referenced, up to and including 2017. Oscillatory integrals are the subject of ongoing research: Yang and Ma^24^, Zaman et al.^25^ used Levin-based approaches to calculate highly oscillatory Fourier integrals in one- and two-dimensional domains, whereas Wang und Xiang^26^ applied a Levin method to singular integrands. Recent articles from Kayijuka et al.^27^ dealt with oscillatory Fourier integrals with singular integrands, while Zaman et al.^28^ used a Levin based approach to evaluate the integrals over Bessel functions. For integrals containing Bessel functions^29^, alternative methods are sometimes expedient: for example the transformation to a contour integral in the complex plane, which is then exponentially damped in an asymptotic manner. The previously mentioned path is pursued by this work in order to numerically calculate the oscillating integral in the Coulomb propagator.

In the following method sections the Coulomb propagator is calculated according to three procedures:

1. Gauss–Pollaczek quadrature along the real axis
2. Gauss–Legendre quadrature along the real axis
3. Transformation into a complex contour integral with exponential damping and numerical calculation using a Gauss–Legendre quadrature.

The paper is structured as follows: first the Coulomb propagator in the L$^2$ representation of Sturmian functions is reviewed. Then the the Gauss–Pollaczek quadrature is explained and numerically stable methods for the calculation of the Pollaczek polynomials are shown. Thereafter the transformation of the continuum part into a contour integral in the complex plane is explained and the calculation of the discrete part of the Coulomb propagator is presented. The result section then demonstrates a comparative analysis of the three integration methods. In the last section potential applications are discussed.

## Coulomb propagator in the L$^2$ representation

The Coulomb propagator $K( \mathbf {r}\,', t'; \mathbf {r}, t )$ solves the time-dependent Schrödinger equation using a $\delta$-function as the inhomogeneity, while $\mathbf {r}$ symbolizes the space coordinate and *t* time.

2 $$\begin{aligned} \left[ i \frac{\partial }{\partial t'} \;\; - \;\; H_0 (\mathbf {r}\,')\right] \; K^+ (\mathbf {r}\,' , t' ; \mathbf {r} , t) \; = \; i ~\delta (\mathbf {r}\,' - \mathbf {r}\,)~ \delta (t' - t) \end{aligned}$$

with $$t' > t$$ and

3 $$\begin{aligned} H_0(\mathbf {r}\;\!) \; = - \frac{1}{2} {\mathbf {\nabla }_r}^2 \;\; - \;\; \frac{z}{r} \end{aligned}$$

where *z* symbolizes the effective nuclear charge.

Since the Hamilton operator $H_0$ is time-independent, the Coulomb propagator is only a function of $t'-t$ in the time part. Thus, $K(\mathbf {r}\,', t'; \mathbf {r}, t )$ is also a solution for the following:

4 $$\begin{aligned} \left[ - i \frac{\partial }{\partial t} \;\; -\;\; H_0 (r) \right] K^+(\mathbf {r}\,' , t' ; \mathbf {r} , t) \; = \; i ~\delta (\mathbf {r}\,' - \mathbf {r}\,) ~\delta (t' - t) \end{aligned}$$

In the time domain $K(\mathbf {r}\,', t'; \mathbf {r}, t)$ satisfies the initial condition:

5 $$\begin{aligned} K(\mathbf {r}\,', t; \mathbf {r}, t) \; = \; \delta (\mathbf {r}\,' - \mathbf {r}) ~~~~ \text {for} ~~t' = t \end{aligned}$$

The probability of the system to be in state $\Psi (\mathbf {r}\,',t')$ – under the condition described by $\Psi (\mathbf {r}, t_i)$ at time $t_i$—can therefore be depicted as an integral through all possible paths throughout the configuration space.

6 $$\begin{aligned} \Psi (\mathbf {r}\,' , t') \; = \; \int d^3 \mathbf {r} ~~ K^+ ( \mathbf {r}\,' , t'; \mathbf {r} , t_i ) ~\Psi (\mathbf {r} , t_i) \end{aligned}$$

For the Coulomb propagator, a partial wave expansion can be conducted in accordance with angular momentum eigenstates; the physical solution of the stationary radial Schrödinger equation to the angular momentum *l* and energy $E = k^2/2$ is as follows:

7 $$\begin{aligned} \left( - \frac{1}{2} \frac{d^2}{dr^2} \;+ \; \frac{l(l+1)}{2 r^2} \; - \; \frac{z}{r} \; - \; E \right) ~ \Psi _l^+(r,E) \; = \; 0 \end{aligned}$$

$\Psi _l^+$ are given by^30^

8 $$\begin{aligned} \Psi _l^+ (r, E) \; = \; \sqrt{\frac{1}{2\pi k}} ~\, i^l \, ~ \frac{F_l^c (r,k)}{J_l^+ (k)} \end{aligned}$$

with

9 $$\begin{aligned} F_l^c (r , k) \; = \; r^{l+1} \; e^{i kr} ~\; M\!(l \! +\!1\!-\!i \frac{z}{k},\; 2l\!+\!2;\; -2i kr) \end{aligned}$$

Here, *M*(*a*, *b*, *x*) is a confluent hypergeometric function and $J^+_l(k)$ is the Jost function, which is given by:

10 $$\begin{aligned} J_l^+ (k) \; = \; i^l \; \frac{e^{-\pi \frac{z}{2k}} ~\; \Gamma \!(2l\!+\!2)}{(2k)^{l+1} ~\; \Gamma \!(l\!+\!1 \!-\! \frac{i z}{k})} \end{aligned}$$

A factor $( \frac{1}{2k\pi } )^{\frac{1}{2}}$ occurs due to normalization to an energy delta function as follows:

11 $$\begin{aligned} \int r^2 \mathrm {d} r ~~ \Psi _l^+ (r,E) ~\; \Psi _l^{+*} (r,E' ) \; = \; \delta (E - E') \end{aligned}$$

The root of the Jost function is given by the Gamma function if the argument $l+1-i~ z/k$ results in a negative integer.

The Coulomb spectral function is defined as the inverse of the square of the absolute value of the Jost function:

12 $$\begin{aligned} C_l(k) \; = \; [ J_l^+ \!(k) \; ( J_l^+ \!(k) )^* ]^{-1} \; = \; e^{\pi \frac{z}{k}} ~ (2k)^{2l+2} ~ \frac{\Gamma (l \!+\!1 \!- \! \frac{i z}{k}) \; ~ \Gamma (l\!+ \!1 \!+ \! \frac{i z}{k})}{ [\Gamma (2l \! + \!2) ]^2} \end{aligned}$$

Consequently, the Coulomb propagator can be depicted as a Stieltjes integral *via* the Coulomb spectral function.13

The $Y_{lm}$ within are spherical harmonics that encapsulate the angular part.

**Table 1:** Values for Pollaczek polynomial $p_{14}^{12}$ with different calculation schemes.

${\lambda }$	Number of digits	Recursion (36)	Horner scheme
2.0	10	1.742914088 E9	1.742914132 E9
1.5	10	8.451334338 E8	8.451334461 E8
1.5	15	8.4513344511522 E8	8.4513344511522 E8
1.0	10	1.962234937 E8	1.96223497 E8
0.5	10	2.049468492 E6	2.04946864 E6
0.25	10	78.0	28.3818683
0.25	15	28.38178229	28.381868292538
0.2	10	125.9999999	6.22356038 E-3
0.2	15	0.107611721	6.2235603856247 E-3
0.2	20	6.223619 E-3	6.22356038562481321 E-3
0.17	10	4.3460064 E7	8.90536721 E-9
0.17	15	− 2592.0	8.9053671758903 E-9
0.17	20	− 0.086541048	8.905367175890381173 E-9
0.17	25	− 4.68 E-7	8.905367175890381024 E-9
0.17	30	8.924151941 E-9	8.905367175890381024 E-9
0.17	35	8.905367077999999 E-9	8.905367175890381024 E-9
0.155	15	1.83768 E23	1.556721695 E-24
0.155	20	1.83768 E15	1.5567216955997484 E-24
0.155	30	−2695264.0	1.556721695599748882 E-24
0.155	40	2.14768 E-4	1.556721695599748882 E-24
0.155	50	− 7.44000000000 E-16	1.556721695599748882 E-24
0.155	60	1.4970000000000 E-24	1.556721695599748882 E-24
0.155	70	1.5567216954857 E-24	1.556721695599748882 E-24

Computation of $p_{14}^{12}(x)$ with $E = - \frac{1}{2 {n_b}^2} ,$ $n_b =13$, $\ z=1$. Column 1: scaling parameter $\lambda$, column 2: precision, number of digits, column 3: computation of $p_{14}^{12}$ with recursion formula (36), column 4: computation of $p_{14}^{12}$ using the Horner scheme (38).

Here, the bound states occur at poles in the spectral density at $$E_{n_b} = -z^2/2n_b^2 $$, where $$n_b = n_r+l+1$$ is the full quantum number and $$n_r \in [0,1,\ldots ]$$ is the radial quantum number. Then the residue of the integrand at each bound-state energy is given by

14 $$\begin{aligned} \Psi _{E_{n_b}} \!(r) ~~ \Psi ^*_{E_{n_b}}\!(r') \; e^{-iE_{n_b}(t'-t)} \ = \ -2\pi i \lim _{E\rightarrow E_{n_b}} \left[ \frac{E-E_{n_b}}{2\pi k} \; \; C_l(k) \; \frac{F_l^c(r,k)}{r}\frac{F_l^{c*}(r',k)}{r'} \; e^{-iE(t'-t)}\right] \end{aligned}$$

where the normalized bound-state wavefunction is

15 $$\begin{aligned} \Psi _{E_{n_b}}(r) = \sqrt{\frac{zn_r!}{(n_r \! + \!2l \! + \! 1)!}} ~ \; \frac{1}{n_b ~ r} \left( \frac{2z r}{n_b}\right) ^{l+1} \; e^{-\frac{zr}{n_b}} \; L^{2l+1}_{n_r} \!\! \left( \frac{2zr}{n_b}\right) \end{aligned}$$

with $L^{2l+1}_{n_r}$ a Laguerre polynomial. As shown in^15^, the origin regular solution $\Psi _l^+(r, E)$ to positive energy can be developed on an L$^2$ basis as follows:

16 $$\begin{aligned} \Psi _l^+ (r,E) \; = \; \Psi _0^{l+} (k,\lambda ) ~ \; \displaystyle \sum _{n=0}^{\infty } \; \Psi _n^l \! (k,\lambda ) \; ~ \phi _n^l \!(r,\lambda ) \end{aligned}$$

with square-integrable functions $\phi _n^l$ given by:

17 $$\begin{aligned} \phi _n^l (r,\lambda ) \ = \ (\lambda r)^{l+1} ~ e^{-\lambda \frac{r}{2}} ~ L_n^{2l+1}(\lambda r) \end{aligned}$$

**Table 2:** Bound states sum.

$${n_{max}}$$	Direct computation with upper limit $${n_{max}} $$	N	$${\Delta }$$	Aitken–Neville
100	− 1.83169083048 E6	20	5	− 1.81619528194 E6
	− i 1.12479990209 E4			− i 1.12476163284 E4
200	− 1.82010746648 E6	20	10	− 1.81619528194 E6
	− i 1.12476406444 E4			− i 1.12476163284 E4
300	− 1.81793848764 E6	20	15	− 1.81619528194 E6
	− i 1.12476211534 E4			− i 1.12476163284 E4
400	− 1.81717695960 E6			
	− i 1.12476178582 E4			
500	− 1.81682396089 E6			
	− i 1.12476169558 E4			
600	− 1.81663204447 E6			
	− i 1.12476166312 E4			
700	− 1.81651626034 E6			
	− i 1.12476164919 E4			
800	− 1.81644108249 E6			
	− i 1.124761642431 E4			
10,000	− 1.81619685703 E6			
	− i 1.12476163284 E4			

Computation of the sum over the discrete spectrum $\displaystyle \sum _{n_b = l+1}^{n_{max}} f_{n_b}^l = 2\pi i \displaystyle \sum _{n_b} ~^{\quad Res}_{l+1-\frac{iz}{k} \rightarrow -n_b} \left[ ~ \frac{\rho _l(x)}{1-x} ~ p_{n}^l(x) ~ p_{n'}^l(x) ~ e^{-iE(x)(t'-t)}\right]$ see (48) with $l=4$, $n=5$, $n'=2$, $z=1$, $\lambda = 0.41$, $t'-t=1$. Column 1: upper summation bound, column 2: values achieved using direct summation, column 5: values achieved using extrapolation Aitken–Neville (51), column 3,4: parameters used in extrapolation (50), (51).

The variable $\lambda$ is a scaling parameter obeying the restriction $\lambda > 2z / (l+1)$. Larger values of $\lambda$ result in maxima of $\phi _n^l$ that are further from the origin.

**Table 3:** Results for angular momentum $l=0$.

$\varvec{(t'-t)}$	Number abscissae	Gaus–Pollaczek	Real axis Gaus-Legendre	Complex plane Gauss-Legendre
1.0 E-80	10	2.727272727	6.001279383	9.642208437
		+ i 0.0	+ i 4.62365 E-77	+ i 4.811958978
	20	4.285714285	6.000000000	6.044227103
		− i 2.45918 E-78	+ i 1.88072 E-76	− i 0.00374479340
	40	5.121951219	6.0	6.00000008
		− i 6.77061 E-78	− i 7.49090 E-76	− i 9.153625010 E-8
	80	5.555555555	6.0	6.000000000
		− i 1.594694 E-77	− i 2.977681177 E-75	− i 3.626107 E-20
	160	5.443796163		6.0
		− i 1.336909 E-77		− i 3.6 E-26
	400	5.910224438		
		− i 9.2337 E-77		
	800	5.955056179		
		− i 1.887961247 E-76		
	1200	5.970024979		
		− i 2.854644922 E-76		
0	Analytical result from equation (45): 6.0			
0.005	10	2.71276022	3.906879828	7.137614255
		+ i 0.3001158573	− i 2.9710717034	+ i 3.218625959
	20	4.06950003	3.739538067	3.476610968
		− i 1.201517149	− i 2.8202986477	− i 1.667040419
	40	3.339719838	3.944706544	3.4258073217
		− i 2.389991495	− i 1.7066616554	− i 1.6694750461
	80	4.449667515	2.89958992955	3.425753490
		− i 2.099578058	− i 1.88908157	− i 1.669519791
	160	3.850151307	3.4111402145	3.4257534258
		− i 1.926234134	− i 1.7085039394	− i 1.6695198538
	400	3.5090977634	3.4340355141	3.4257534258
		− i 1.4721667528	− i 1.7709989833	− i 1.6695198538
	800	3.5186221508	3.435689731	3.4257534258
		+ i 1.7892551172	− i 1.687052360	− i 1.6695198538
	1200	3.2479752935	3.472742772	
		+ i 1.5476880522	− i 1.699163777	
	2400		3.4421350323	
			− i 1.6433753013	
0.5	10	− 3.477793445	− 1.193293649	1.4056819897
		+ i 7.4617299928e-1	+ i 2.4938006850	+ i 4.9888146679
	20	− 3.6238167665	2.2984267581	− 0.32440141493
		− i 1.2560908355	+ i 0.9952370052	+ i 0.7072393403
	40	− 1.3465166080	− 0.33693804687	− 0.36308982742
		+ i 2.3744873077	+ i 1.3963439575	+ i 0.70040133009
	80	− 1.0670328810	0.60668182561	− 0.3630899216
		+ i 1.1506035351	+ i 0.68321429432	+ i 0.7004013931
	160	− 1.333233898	0.04131505033	− 0.3630899216
		+ i 2.5758824241	+ i 0.8840672501	+ i 0.7004013931
	400	2.251787747	− 0.32472379908	− 0.3630899216
		+ i 5.5177208499	+ i 0.22285013492	+ i 0.7004013931
	800	− 0.1456923378	− 0.3315742524	
		+ i 0.4962831499	+ i 0.5914511421	
	1200	0.39014559103	− 0.21717058189	
		+ i 0.61176814878	+ i 0.7197565876	
	2400		− 0.4871636252	
			+ i 0.6112139315	
	4800		− 0.3207608876	
			+ i 0.66714051745	
5.0	10	1.2582899589	6.686196571	− 0.33116862071
		− i 2.5458362767	+ i 1.021407132	+ i 0.17751464863
	20	− 0.9928173236	2.777628488	− 0.02381465223
		− i 3.104949757	+ i 1.023771144	− i 0.068906063706
	40	− 1.8975170441	1.3727591716	− 0.015493774118
		−i 0.88522225699	− i 2.2268802507	− i 0.07606821836
	80	− 2.398165120	− 0.6727684468	− 0.015493764931
		− i 0.65455487666	− i 0.2733979867	− i 0.07606828280
	160	− 0.051590316113	− 0.6695234503	− 0.01549376493
		+ i 0.96934350202	− i 0.2057220436	− i 0.07606828280
	400	− 1.4129548818	− 0.1569182666	− 0.01549376493
		+ i 4.189284105e-1	+ i 0.32271066527	− i 0.07606828280
	800	0.1621209990	0.09060575377	
		− i 0.13152919497	− i 0.1956157272	
	1200	− 0.06628726465	0.04020166145	
		+ i 0.079199787944	− i 0.08079464576	

Numerical values of the Integral (52) $$\begin{aligned} \sum\kern -22.5pt\int \it{dx} ~ \frac{\rho _l(x)}{1-x} ~ p_{5}^l ~ \; p_{2}^l ~ e^{-iE(x)(t'-t)} \end{aligned}$$ with l = 0 for different values of $t'-t$, (z=1, $\lambda$ = 2.01), evaluated using the Gauss–Pollaczek quadrature (column 3), Gauss–Legendre quadrature (column 4) along the real axis and as a contour integral with the Gauss–Legendre quadrature (column 5) and, in column 4 and 5, the value of the sum over the bound states is added to enable comparison with column 3.

The great advantage of the expansion (16) lies in the factorization of $\Psi _l^+$ in an energy independent spatial part $\phi _n^l$ and a kinetic part, exclusively contained in the expansion coefficients $\Psi _0^{l+} ~ \Psi _n^l$. These are representative in an analytical way and consist of the Pollaczek polynomials $p_n^l$.

18 $$\begin{aligned} \Psi _n^{l}(k,\lambda ) \; = \; p_n^l(k,\lambda ) ~ \frac{n! (2l+1)!}{(n+2l+1)!} \end{aligned}$$

The function $\Psi _0^{l+}$ is independent of n and given by:

19 $$\begin{aligned} \Psi _0^{l+} \; = \; \frac{i^l}{J_l^+ (k)} ~ \frac{(2\sin \gamma )^{l+1} ~ \xi ^{\frac{i z}{k}}}{( \sqrt{2\pi k} ~ \; (2k)^{l+1}} \end{aligned}$$

and $\gamma$, $\xi$ and *x* are defined by

$$\begin{aligned} \sin \gamma = \sqrt{1-x^2} = \frac{k\lambda /2}{E+\lambda ^2/8}, \xi = \frac{\lambda +2ik}{\lambda -2ik} = e^{i\gamma }, x = \frac{E-\lambda ^2/8}{E+\lambda ^2/8} = -\cos \gamma \end{aligned}$$

An expansion of the irregular Coulomb function scaled at the origin similar to $r^{-l-1}$ cannot be realized using the L$^2$ functions $\phi _n^l$. However, an expansion according to $\phi _n^l$ and showing the same asymptotic behavior as the irregular Coulomb function in the limit $r\rightarrow \infty$, can be realized. This finite solution at the origin—behaving like the irregular Coulomb function for large *r* and being normalized to an energy delta function—is given by:

20 $$\begin{aligned} \Psi _l^{+ \mathrm {irreg}} (r,E) \; = \; \displaystyle \sum _{n=0}^{\infty } \phi _n^l (r,\lambda ) ~ \; Q_n^{l+}(k,\lambda ) \end{aligned}$$

with the expansion coefficients $Q_n^{l+}$, which themselves consist of the Pollaczek functions $q_n^{l+}$^15^:

21 $$\begin{aligned} Q_n^{l+} \; = \; \frac{\lambda ~ q_n^{l+} \quad n!}{2 \pi \;\;(E \! + \! \frac{\lambda ^2}{8}) \;\; (n\!+\!2l\!+\! 1)!~ \;(2l\!+\!1)!~ \; \psi _0^{l+}} \end{aligned}$$

Following transformation to the variable $x (E) = ( E - \lambda ^2 /8 ) / ( E + \lambda ^2 /8 )$ by which the semiinfinite energy interval $[0, \infty ]$ transforms to the x interval $[-1, +1]$ and the insertion of (16), (18), (19), and (10) into (13), results in the L$^2$ expansion of the Coulomb propagator according to the basis functions $\phi _n^l$ :

**Table 4:** Results for angular momentum $l=1$.

${(t'-t)}$	Number abscissae	Gaus–Pollaczek	Real axis Gaus–Legendre	Complex plane Gauss–Legendre
1.0E-80	10	32.16783216	39.97558703	− 96.15945597
		+ i 9.071954195 E-79	− i 3.590626976 E-78	− i 26.67315045
	20	38.73517786	40.00000009	38.75984423
		− i 1.937058241 E-78	− i 3.590650999 E-78	+ i 0.3920371993
	40	39.81849120	40.0	39.99999227
		− i 2.669846775 E-78	− i 3.590651000 E-78	− i 3.574273123 E-6
	80	39.97562063	40.0	40.0
		− i 3.104303637 E-78	− i 3.59065100 E-78	+ i 3.793870810 E− 18
	160	39.99683866	40.0	40.0
		− i 3.34065185 E-78	− i 3.590651000 E-78	− i 6.119621 E-30
	400	39.99979311	40.0	40.0
		− i 3.488967329 E-78	− i 3.590651000 E-78	+ i 7.9 E-35
	800	39.99997394	40.0	
		− i 3.539524561 E-78	− i 3.590651000 E-78	
0	Analytical result from equation (45): 40.0			
0.005	10	32.16508112	39.81579038	− 96.27028870
		− i 0.4535865655	− i 1.551637521	− i 28.26187564
	20	38.71809664	39.80022701	38.53795792
		− i 0.9682731062	− i 1.549782365	− i 1.119113023
	40	39.7592980	39.79179955	39.77797257
		− i 1.331749104	− i 1.509772520	− i 1.510166952
	80	39.82089624	39.773359859	39.77798247
		− i 1.521874883	− i 1.512795302	− i 1.510165622
	160	39.76186869	39.77775388	39.77798247
		− i 1.495753805	− i 1.511171641	− i 1.510165621
	400	39.78077531	39.77793391	39.77798247
		− i 1.518514989	− i 1.510466632	− i 1.510165621
	800	39.77753565	39.77798989	39.77798247
		− i 1.513764163	− i 1.510114361	− i 1.510165621
0.5	10	7.897616860	8.698416257	− 124.4072348
		− i 35.02119019	− i 35.69875031	− i 58.56579560
	20	9.225592473	4.849746595	− 0.5995298871
		− i 17.82815360	− i 21.88044169	− i 24.47342905
	40	2.927106852	− 0.6532723583	0.6238724682
		− i 29.6969524054	− i 23.82319647	− i 24.77194207
	80	1.098644464	1.178538620	0.6238798285
		− i 27.28458753	− i 24.99715298	− i 24.77193806
	160	− 0.3092012308	0.6541107487	0.6238798285
		+ i 25.00630721	− i 24.59319531	− i 24.77193806
	400	0.8275983323	0.6352292771	0.6238798285
		− i 24.75240185	− i 24.83153546	− i 24.77193806
	800	0.5592836214	0.6287940824	
		− i 24.65709199	− i 24.76213471	
5.0	10	23.59010389	− 20.17827124	− 45.02312630
		+ i 26.19330617	+ i 46.39567912	− i 32.44370521
	20	− 6.585995437	30.68349351	− 2.125151775
		+ i 5.616470441	+ i 31.86569833	+ i 15.51521636
	40	9.710645643	-4.936754757	− 1.253811147
		+ i 4.903124332	+ i 12.000214328	+ i 15.84221490
	80	− 7.364704256	− 6.5893005245	− 1.253807732
		+ i 15.866035128	+ i 5.20745352	+ i 15.84222128
	160	-6.407543593	− 1.707542635	− 1.253807732
		+ i 15.89062954	+ i 14.72359189	+ i 15.84222128
	400	− 0.01881118212	− 0.9528009615	− 1.253807732
		+ i 16.82616727	+ i 15.98141809	+ i 15.84222128
	800	− 1.556539404	−1.218243190	
		+ i 15.96489516	+ i 15.76062352	

Same as Table 3 but with l = 1.


22


Upon closer inspection, the endpoint singularity occurring in the integrand at $x = 1$ is of shape $(1-x)^{l-1/2}$ and can be eliminated through transformation to variable *k*, which is linked to $x = (k^2 - \frac{\lambda ^2}{4}) / (k^2 + \frac{\lambda ^2}{4})$. (In the following the integration bounds for the *x* integration will be omitted, in all formulas it is implicitely assumed that $x \in [-1, 1]$.)

For a wave function $\Psi (\mathbf {r},t)$, an expansion into an orthonormalized function system can be pursued (for simplicity, index m is omitted in the following equations):

23 $$\begin{aligned} F_n^l (\mathbf {r}\,) = Y_{lm} ({\hat{r}}) ~ \frac{f_n^l (r)}{r} \ ,\ \ \ \ \ \ \ f_n^l(r) = \sqrt{\frac{\lambda n!}{(n+2l+2)!}} ~ (\lambda r)^{l+1} ~ e^{-\lambda \frac{r}{2}} ~ L_n^{2l+2} (\lambda r) \end{aligned}$$

with orthonormalization relation:

24 $$\begin{aligned} \int d^3 \mathbf {r} ~ F_n^l ~ F_{n'}^{l'} \; = \; \delta _{l l'} ~ \delta _{n n'} \end{aligned}$$

Thus, $\Psi (\mathbf {r},t)$ can be expanded in $F_n^l$ with time-dependent expansion coefficients $\alpha _n^l(t)$:

25 $$\begin{aligned} \Psi ( \mathbf {r}, t) \; = \; \displaystyle \sum _{nl} \alpha _n^l (t) ~\; F_n^l (\mathbf {r}\,) \end{aligned}$$

The insertion of (25) into the conditional equation (6) and a projection with $F_n^l(\mathbf {r}\,)$ then leads to26

In (26), the integration over x also includes the sum over the bound-state spectrum.

**Table 5:** Results for angular momentum $l=4$.

${(t'-t)}$	Number abscissae	Gaus–Pollaczek	Real axis Gaus–Legendre	Complex plane Gauss–Legendre
1.0E-80	10	4339081.114	4206203.745	− 75722959.05
		i 6.470071133 E-75	− i 7.354765343 E-75	+ i 68277873.89
	20	4434313.403	4435225.036	− 269019.8701
		− i 7.331784442 E-75	− i 7.366713631 E-75	+ i 120307.0401
	40	4435195.678	4435199.999	4435837.369
		− i 7.366031030 E-75	− i 7.366716000 E-75	+ i 57.12640763
	80	4435199.986	4435200.0	4435199.999
		− i 7.366707165 E-75	− i 7.366716 E-75	+ i 4.31435208 E-7
	160	4435199.999	4435200.0	4435200.0
		− i 7.366715910 E-75	− i 7.366716 E-75	+ i 8.602516 E-24
	400	4435199.999	4435200.0	4435200.0
		− i 7.366715999 E-75	− i 7.366716 E-75	+ i 1.1247621 E-76
	800	4435199.999	4435200.0	
		− i 7.366715999 E-75	− i 7.366716 E-75	
	1200	4435199.999	4435200.0	
		− i 7.366715999 E-75	− i 7.366716 E-75	
0	Analytical result from equation (45): 4435200.0			
0.5	10	4329776.444	4189832.724	− 75018398.07
		− i 323313.8133	− i 366687.5194	+ i 6.611314045E7
	20	4418926.488	4418891.004	− 250935.6554
		− i 365896.2260	− i 367282.9684	− i 303101.8894
	40	4418883.856	4418865.623	4419500.030
		− i 367301.6250	− i 367282.9960	− i 367219.0111
	80	4418866.422	4418865.627	4418865.626
		− i 367282.2810	− i 367283.0198	− i 367283.0188
	160	4418865.533	4418865.626	4418865.626
		− i 367283.0351	− i 367283.0187	− i 367283.0188
	400	4418865.623	4418865.626	4418865.626
		− i 367283.0218	− i 367283.0188	− i 367283.0188
5.0	10	3438490.295	2968516.038	− 69428885.15
		− i 3049251.161	− i 3114395.656	+ i 49076167.77
	20	3172112.773	3190481.291	− 1151665.312
		− i 3109310.235	− i 3116836.069	− i 3517891.112
	40	3190680.842	3189536.315	3190212.609
		− i 3113572.463	− i 3116126.281	− i 3115980.325
	80	3189851.424	3189603.014	3189607.812
		− i 3116231.449	− i 3116105.397	− i 3116103.558
	160	3189619.174	3189607.705	3189607.812
		− i 3116076.000	− i 3116103.843	− i 3116103.558
	400	3189606.267	3189607.820	3189607.812
		− i 3116102.866	− i 3116103.557	− i 3116103.558

Same as Table 3 but with l = 4 and ( z = 1, $\lambda$ = 0.41).

Weight $\rho _l(x)$ is given by

27 $$\begin{aligned} \rho _l (x) \; = \; (2\sin \gamma )^{2l+1} ~ e^{\frac{\pi z}{k}} ~ \xi ^{2i \frac{z}{k}} ~ \; \Gamma (l\!+\!1 \! - \! \frac{iz}{k}) ~ \; \Gamma (l\!+\!1\!+\! \frac{iz}{k}) \end{aligned}$$

with poles when the argument of the $\Gamma$ function is either zero or a negative integer, and $N_n^l$ by

28 $$\begin{aligned} N_n^l \; = \; \frac{n!}{(n+2l+1)!} \end{aligned}$$

The integration in the configuration space of matrix elements $\langle \phi _{n''}^l | f_n^l \rangle$ and $\langle \phi _{n'}^l | f_k^l \rangle$ can be achieved analytically and is only non zero for $n''=n, n+1$ and $n'=k, k+1$. Consequently, the sum over $n''$, $n'$ reduces to only a few terms when using the expansion (25). A remaining challenge is to solve the oscillating integrals:29and to determine the infinite sum over the bound states. In the second line of (29) the first term describes the continuum part. The discrete part over the bound states can be written in accordance with (14) as the negative sum over all residues of the weight function $\rho _l$ evaluated at the poles of the $\Gamma$ function.

For $n'', n' \in [0, N-1]$ the number of integrations scales with $N^2$ for each angular momentum *l*, and can be drastically reduced by exploiting the three-term recursion relation (see 32) for the Pollaczek polynomials:

30 $$p_{n'+1}^l \; p_n^l \; = \;\frac{1}{n'\!+\!1} \; \bigg [ \frac{b_{n'}^l}{b_n^l } (n\!+\!1) \; p_{n'}^l p_{n+1}^l \; + \; \frac{4z}{\lambda }  \times \big ( 1\!-\! \frac{b_{n'}^l}{b_n^l} \big ) \; p_{n'}^l p_n^l \; + \; \frac{b_{n'}^l}{b_n^l} ( n\!+\!2l\!+\!1) \;p_{n'}^l p_{n-1}^l \; - \; (n' \!+\!2l\!+\!1) \; p_{n'-1}^l p_{n}^l \bigg ]$$

with $$b_n^l = -2(n+l+1-2z/ \lambda ) $$

This recursion directly applies to (29). For calculation of the square matrix $$I_{n' n}^l $$ for $$n', n \in [0, N-1]$$, it is necessary to compute the 2 N-1 start values $$I_{0 n}^l$$ in n, which recur with (30) upwards in $$n'$$. First test cases give evidence that the recursion is stable. This is further supported by the fact that the diagonal elements $$I_{n n}^l$$, which are greater than the off-diagonal elements, are constructed from much smaller values $$I_{0 n}^l$$.

In the following sections, we investigate integrals $I_{n' n}^l$ without restriction for integer values $n, n' \ge 0$. Integration *via* the continuum is examined through three different methods: the Gauss–Pollaczek and Gauss–Legendre quadratures along the real axis and calculations with a rotation into the complex plane, resulting in exponential damping. For this purpose, the Pollaczek polynomials in the Coulomb propagator are discussed and their numerical calculation is introduced in greater detail in the following section.

## Gauss–Pollaczek quadrature and calculation of the Pollaczek polynomials

The Pollaczek polynomials are real for real arguments and form an orthogonal function system with regard to the weight function $\rho _l(x)$ specified in (27):31

The integrand occurring in (29) differs from (31) only in terms of the factor $e^{-iE(t'-t)} / (1-x)$; therefore, it can be assumed that a Gauss–Pollaczek quadrature is well-suited for calculating (29); this hypothesis is examined in more detail in the following sections.

The Pollaczek polynomials obey, similarly to orthogonal polynomials, a three-term recursion relationship as follows:

32 $$\begin{aligned} (n\!+\!1) \; p_{n+1}^l - 2 \left[ (n\!+\!l\!+\!1\!-\! \frac{2z}{\lambda })x + \frac{2z}{\lambda } \right] \; p_n^l + (n\!+\!2l\!+\!1) \;p_{n-1}^l \;= \; 0 \end{aligned}$$

The orthogonality relationship of the Pollaczek polynomials enables the development of an N-Point Gauss quadrature that exactly determines integrals whose integrands contains the Coulomb spectral function as follows:33$x_j,j=1,\ldots ,N$ are the *N* roots of the Pollaczek polynomial $p_N^l$ and $w_j,j=1,\ldots ,N$, with corresponding weights.

The great advantage of the Gauss Pollaczek quadrature is its inherent feature to include the infinite bound state sum directly in the quadrature method, thus avoiding the separate evaluation. This is also reflected by the fact, that the zeros of the Pollaczek polynomials occur at positive as well as at negative energies. Therefore the sum on the right side of (33) approximates both parts- discrete and continuum.

Both the weights and the roots can be calculated very easily *via* diagonalization of a tridiagonal matrix whose coefficients are given by the monic recursion relation of the (orthonormal) Pollaczek polynomials:

34 $$\begin{aligned}&\frac{1}{2} \left[ \frac{(n\!+\!1) \;(n\!+\!2l\!+\!2)}{(n\!+\!l\!+\!1\!-\! \frac{2z}{\lambda }) \; (n\!+\!l\!+\!2 \!-\! \frac{2z}{\lambda })}\right] ^{\frac{1}{2}} p_{n+1}^l - \left[ \frac{\frac{2z}{\lambda }}{(n\!+\!l\!+\!1\!-\!\frac{2z}{\lambda })} -x \right] p_n^l \nonumber \\&\quad + \frac{1}{2} \left[ \frac{n \; (n\!+\!2l\!+\!1)}{(n\!+\!l\!+\!1\!-\!\frac{2z}{\lambda })(n\!+\!l\!-\!\frac{2z}{\lambda })} \right] ^{\frac{1}{2}} p_{n-1}^l \; = \; 0 \end{aligned}$$

The weights in (33) result from the first component of the ith eigenvector $v_{1i}$ of a tridiagonal matrix together with the zeroth moment, which are thus given by:

35 $$\begin{aligned} w_i \; = \; \frac{v^2_{1i}}{(2l+1)!} ~ \; (l+1-\frac{2z}{\lambda }) \end{aligned}$$

At this point, the following question arises: how well does this Gauss–Pollaczek quadrature work for integrals of the form (29) containing terms $e^{-iE(t'-t)} / (1-x)$, which oscillate strongly? This question is examined and answered in more detail in the following section.

For the analysis of (29), the Pollaczek polynomials must be calculated for both negative discrete and positive values of *E* in the interval $[ 0, \infty ]$.

The sum over the bound-state spectrum—here, the sum over all residues of the integrand—occurs only when parameter *z*—the atomic number—is positive. Residues result from the poles of the Gamma function when the argument $l+1-iz/k$ is a negative integer and characterize the bound states at $E_{n_b} = -z^2 / 2n{_b}^2$.

If parameter $\lambda$ is chosen in such a way that $\lambda = 2z / (n_r+l+1)$ is valid, then $\phi _{n_r}^l (r, \lambda )$ is directly proportional to a bound eigenstate. This value of $\lambda$ can thus be used to optimize the wave function of a particular eigenstate.

As stated in^15^, the three-term recursion relationship (32) can be used as a starting point for calculation.

The division of all terms in (32) by $p_{n}^l$ setting $p_{n+1}^l = r_{n+1}^l p_{n}^l$ with $1 / r_{0}^l :=0$ leads to the following recursion:

36 $$\begin{aligned} r_{n+1}^l \; = \;\frac{1}{n+1} \left[ 2\; (n\!+\!l\!+\!1) \; x \;+ \; \frac{4z}{\lambda }(1-x) \; - \; \frac{(n\!+\!2l\!+\!1)}{r_n^l} \right] \end{aligned}$$

As shown by calculations, this algorithm is numerically stable for positive energy values $x \in [-1, +1]$ and any values of $\lambda$.

For the discrete part corresponding to negative energies $E_{n_b} = -z^2 / 2n_b^2$ and $\lambda \rightarrow 2z / ( n_r+l+1)$, however, the recursion (36) becomes numerically unstable for $r_{n+1}^l$; these values, however, are needed for (29).

At this point, it is best to express the Pollaczek polynomials *via* a hypergeometric function and implement their evaluation *via* the Horner scheme as follows:

37 $$\begin{aligned} p_n^l (x,\lambda ) \; = \; \frac{(n\!+\!2l\!+\!1)!}{(2l\!+\! 1)! ~n!} ~ (-\xi )^n ~ \;_2 F_1 (-\!n, \; l\!+\!1\!-\!\frac{i z}{k};\; 2l\!+\!2; \; 1\!-\! \frac{1}{\xi ^2}) \end{aligned}$$

The $_2 F_1$ reduces to a polynomial in $1-1/\xi ^2$ due to a negative integer in the first argument. The Horner scheme is a fundamental algorithm to evaluate polynomials with a minimum of arithmetic operations and is numerically stable. This makes the algorithm best suited for computer implementation.

For the bound states $E_{n_b}=-z^2/2{n_b}^2$, *k* causes purely imaginary and $p_n^l$ results for the following finite sum:

38 $$\begin{aligned}p_n^l (E_{n_b}, \lambda ) \; = \; (n_b \!+ \! 2l \! + \!1)! \; (n_b \! - \! l\! -\! 1)! \; (- \xi )^n \ \displaystyle \sum _{j=0}^{\min ( n , n_b-l-1)} \frac{1}{j!} \times \frac{1}{(n_b \!-\!j)! \;(n_b\!-\!l\!-\!j\!-\!1)! \;(2l\!+\!1\!+\!j)!} \; (1\!-\!\frac{1}{\xi ^2})^j \end{aligned}$$

In Table 1, the values of $p_{14}^{12} (x)$ with $E_{n_b}\!=-\frac{1}{2{n_b}^2}$, $n_b = 13$ were calculated in an exemplary way for different scaling parameter $\lambda$ and with varying accuracy. The values are given in columns 3 and 4: this reflects the calculation with the three-term recursion formula (36) and by calculating the sum (38) according to the Horner scheme.

Implementation is achieved using a numerical package (Java Apfloat) that allows for calculating with user-defined accuracy.

In particular, for small values of $\lambda$ (for $\lambda \rightarrow 2z / (l+1)$), it can be seen that the three-term recursion (36) becomes numerically unstable; the number of digits required for calculation is significantly higher if the same result is sought as with (38). In contrast, calculations using the Horner scheme are stable for all tested values of the scaling parameter.

Consequently, the following assertions can be made:

1. For positive values of *E* or equivalent $x \in [-1, +1]$, the three-term recursion formula (36) is numerically stable.
2. The same applies to bound energies $E_{n_b} = - \frac{z^2}{2{n_b}^2}$ under the condition that $\lambda$ differs from $2z / (l+1)$ in the first two leading digits.
3. For $\lambda \rightarrow 2z / (l+1)$ and $E_{n_b} = - \frac{z^2}{2{n_b}^2}$, the calculation with the Horner scheme is numerically stable.

## Calculation of the continuum part—contour representation

In this section of the paper, the Stieltjes integral part is considered across the positive energy spectrum. To make a clear dsitinction between the whole Stieltjes integral (29)—which includes the bound state sum—the symbol $A_{n n'}^l$ is used to indicate the continuum part:

39 $$\begin{aligned} A_{nn'}^{l} \; = \; \int \limits _{-1}^{+1} dx ~ \frac{\rho _l (x)}{1-x} ~\; p_{n}^l (x) ~ p_{n'}^l (x) ~ e^{-iE(x)(t'-t)} = \int \limits _{0}^{\infty } dE \frac{\rho _l (E)}{E+\lambda ^{2} /8} \; p_{n}^l (E) ~ p_{n'}^l (E) ~ e^{-iE(t'-t)} \end{aligned}$$

For this purpose, a transformation is conducted in the following subsection, resulting in exponential damping. Furthermore a contour representation based on a conformal mapping to the unit circle is explained.

### Exponential damping

Using the connection between the kinetic energy and the absolut value of the impuls, $E=k^{2}/2$, and transformation to the variable *k* given by $x = ( E \!-\! \lambda ^2 /8 ) / ( E\! +\! \lambda ^2 /8 ) = (k^2 \!-\! \frac{\lambda ^2}{4}) / (k^2 \! +\! \frac{\lambda ^2}{4})$ then leads to the following integral:

40 $$\begin{aligned} A_{nn'}^{l} \; = \; \int \limits _0^{\infty } dk ~ \frac{2k}{k^2 + \frac{\lambda ^2}{4}} ~ \rho _l(k) ~ e^{-i \frac{k^2}{2} (t'-t)} ~ p_n^l(k) ~ p_{n'}^l(k) \end{aligned}$$

Here, too, the integrand strongly oscillates. An asymptotic expansion of the integrand identifies the following behavior:

41 $$\begin{aligned} \lim \limits _{k \rightarrow \infty } \; \left[ \frac{2k}{k^2+\frac{\lambda ^2}{4}} ~\; \rho _l (k) ~ e^{-i \frac{k^2}{2}(t'-t)} ~ p_n^l(x) ~ p_{n'}^l(x) \right] \; \sim \; \frac{1}{k^{2l+2}} \end{aligned}$$

This demonstrates that the integrand for large angular momentum numbers *l* is damped to a much greater extent than for small values of *l*. The integrand oscillates widely, especially for large values of k. If traditional integration routines are applied, a more accurate numerical evaluation can only be expected with the use of many abscissae, especially for smaller *l* values.

Consequently, it is necessary to reexpress the integral (39) using a contour integral in the complex plane such that, for the resulting path, the integral is much more strongly damped for large values of *k* than for integration along the real axis.

This is achieved by transformation to the variable *y*: $k = y (1 - i) / \sqrt{2}$.

42 $$\begin{aligned} A_{nn'}^{l} \; = \; -2i \int \limits _0^\infty dy ~ \frac{y \; \;( \frac{\lambda ^2}{4} + i y^2)}{(\frac{\lambda ^2}{4})^2 + y^4} ~ \rho _l (y) ~ e^{- \frac{y^2}{2} (t'-t)} ~ \; p_n^l (y) ~ p_{n'}^l (y) \end{aligned}$$

Thus, the integrand is “practically” free of oscillation with exponential damping.

**Figure 1:**
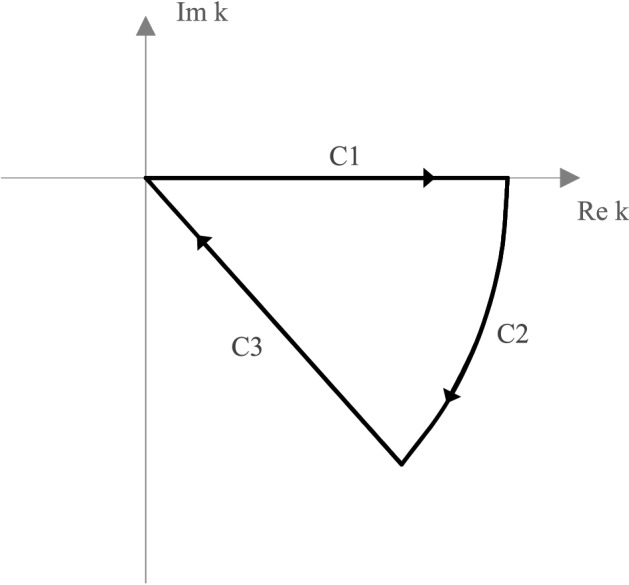
Path in the complex plane required to evaluate the continuum part of the Coulomb propagator. The value of the integral (39) along the real axis is given by the negative sum of the integral along C2 and C3: C1 = –(C2 + C3).

**Figure 2:**
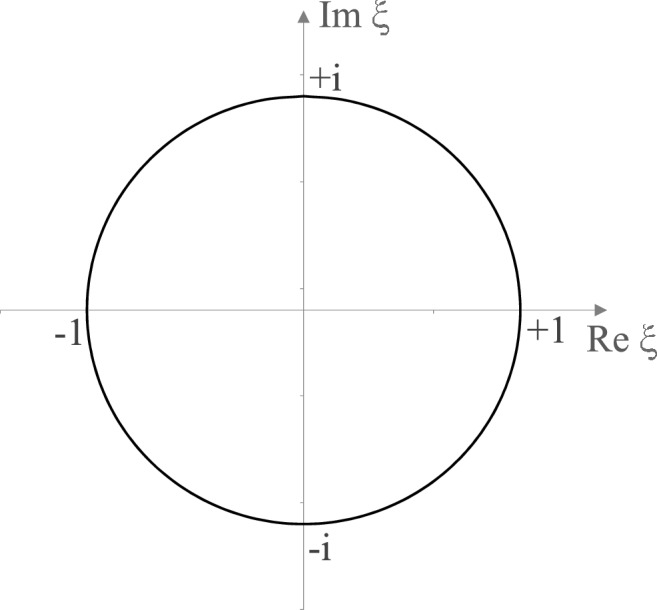
Integration path of the integral over the continuum part (43) after transforming to the complex $\xi$-plane. The integration path is a closed loop along the unit-circle.

The integration path in the complex plane is shown in Fig. 1 and is characterized by a closed path in the lower complex half plane.

In the limiting case of $k \rightarrow \infty$, the contribution to the integral vanishes along the path C$_2$, resulting in the integral $I_{nn'}^{l}$ being determined by the path along C$_3$.

The integral can now be evaluated, for example, with a Gauss–Legendre quadrature, although due to exponential damping, fewer abscissae are necessary than in the integration along the real axis. It is expected that the number of abscissae decreases at higher values of $(t'-t)$.

*Vice versa*, it can be assumed that, for small values of $(t' - t) \approx 0$, the number of abscissae will increase in order to maintain a certain accuracy. However, the numerical input for the calculation with the contour representation in the complex plane is significantly higher since, in such cases, the integrand is evaluated using complex arguments.

### Transformation to unit circle

By transforming to the variable $$\xi = (\lambda + 2ik) / (\lambda - 2ik)$$, a conformal mapping is defined with $$\xi \xi ^* = 1$$. Consequently, previous work has demonstrated the orthogonality of the Pollaczek polynomials. When using their analytical properties^15^ and carrying out the transformation onto the variable $$\xi $$, the integral $$ A_{nn'}^{l}$$ results in the following:43

In the above, $q_n^{l+}$ is the coefficient of the irregular Coulomb function—as shown in (21)—and the integration path is a closed curve over the unit circle in the complex $\xi$ plane, as shown in Fig. 2. Within this closed curve lie the bound states with energies $E = -z^2 / (2{n_b}^2)$ on the real $\xi$ axis; here, the function $q_n^{l+}$ has poles, while $p_n^l$ is free of poles. The integrand has another, higher-order pole at digit $\xi = -1$ due to the argument of the exponential function appearing in the integrand, lying exactly on the integration path, while the poles of $q_n^{l+}$ are located within the unit circle. This contour representation can therefore only be consulted for analysis in the case of $(t' - t) = 0$. According to this behavior, use of the Cauchy principle value and application of the residual theorem enables the integral over the continuum part to be calculated analytically:

44 $$\begin{aligned}\int \limits _{-1}^{+1} dx ~ \frac{\rho _l(x)}{1-x} ~ p_n^l (x) ~ p_{n'}^l (x) \ = \ \frac{2(n_{<} + 2l +1)!}{(2l+1) \; \; n_< !} \ + \ 2 \pi i \sum ~^{\quad Res}_{x(E) \rightarrow x(E_{n_b})} \left[ \frac{\rho (x)}{1-x} ~ p_n^l (x) ~ p_{n'}^l (x) \right] \end{aligned}$$

where $n_<$ specifies the min ($n, n'$). The first term arises from the pole at $\xi =-1$ and the infinite sum over the residues result from the simple bound state poles lying within the closed contour of the unit circle. This sum cancels exactly with the bound states sum in the Coulomb Propagator (29), such that the result for $(t'-t) =0$ is as follows:45

A derivation of the analytical result (45) is given in the supplementary file. The sum in (44) only appears in the case of $z > 0$ and extends across all discrete eigenvalues with a negative value. The sum is convergent, whereas the individual terms are scaled with $1/{n_b}^3$, which is investigated in more detail in the following section.

## Computation of the sum via the discrete spectrum

With analogy to (14), the result for the sum occurring via the discrete spectrum in (29) with (27) is as follows:

46 $$\begin{aligned} -2\pi i \displaystyle \sum _{n_b} ~^{\quad Res}_{l+1-\frac{iz}{k} \rightarrow -n_b} \left[ ~ \frac{\rho _l(x)}{1-x} ~ p_{n}^l(x) ~ \; p_{n'}^l(x) ~ e^{-iE(x)(t'-t)}\right] \end{aligned}$$

The function $\rho _l (x)$ has poles that are given by the $\Gamma$ function $\Gamma (l\!+\!1\!-\!i z/k)$; these occur if the argument results in 0 or a negative number: $l+1- i z/k \; =\; -n_b$

Using the relationships $\Gamma (x) \Gamma (1-x) = \pi / \sin (\pi x)$^31^ and $\sin \gamma = (\xi - 1/\xi ) / 2i$, the residuum occurring in (46) can be calculated. If transformed to the variable $\beta = i z/k$, this results in:

47 $$\begin{aligned} \frac{dx}{d \beta } \; = \; \frac{1}{\beta } \left( \frac{4\beta \; \; \frac{z}{\lambda }}{\beta ^2 - (\frac{2z}{\lambda })^2} \right) ^2 \end{aligned}$$

and evaluation at $\beta = n_b$ in:

48 $$\begin{aligned}&\displaystyle \sum _{n_b} 2 \pi i \quad ^{\quad Res}_{l+1-\frac{iz}{k} \rightarrow -n_b} \quad \left[ \frac{\rho _l}{1-x} ~ p_n^l ~ p_{n'}^l ~ e^{-i E(t'-t)}\right] \; \\& \quad =- \frac{2}{\lambda ^2} (-1)^{2l+1} \displaystyle \sum _{n_b = l+1}^\infty \frac{1}{n_b} \left[ \frac{n_b \!-\! \frac{2z}{\lambda }}{n_b\! +\! \frac{2z}{\lambda }} \right] ^{2n_b}\nonumber \\&\qquad \times \left[ \frac{n_b \; z}{(\frac{2z}{\lambda })^2\! -\! n_b^2 } \right] ^{2l+3} \frac{(n_b \! +\! l)!}{(n_b \!-\!l \!-\!1)!} \; ~ p_n^l (x_{n_b}) ~ p_{n'}^l (x_{n_b}) \left( E_{n_b} \! +\! \frac{\lambda ^2}{8}\right) e^{\frac{i z^2}{2 n_b^2} (t'-t)} \; \equiv \; \displaystyle \sum _{n_b = l+1}^\infty f_{n_b}^l \nonumber \end{aligned}$$

where the Pollaczek polynomials are evaluated at argument

49 $$\begin{aligned} x_{n_b} \; = \; \left( E_{n_b} - \frac{\lambda ^2}{8}\right) / \left( E_{n_b} + \frac{\lambda ^2}{8}\right) , \qquad E_{n_b} = -\frac{z^2}{2 \; n_b^2} \end{aligned}$$

The sum then scales following $1 / {n_b}^3$ and exhibits convergence.

Since the limit of the function for $n_b \rightarrow \infty$ cannot be crossed, computers for numerical evaluation aborted the sum at a certain value. The best choice to yield higher accuracy is to calculate a sequence of partial sums $(S_1, S_2, \ldots S_N)$ and extrapolate these to a limit value $S_\infty$. This is the basic idea of the Aitken–Neville method: It is an algorithm which uses the partial sum values as input and thus realizes a convergence acceleration.

The algorithm itself is defined by the Aitken–Neville tableau: When defining partial sums and the variable $h_k$ as

50 $$\begin{aligned} S_i = \displaystyle \sum _{n_b = l+1}^{i\Delta } f_{n_b} , \qquad h_k = \frac{1}{k \; \Delta } \end{aligned}$$

the Aitken–Neville tableau is given as follows:

51 $$\begin{aligned} T_{i,k} = \frac{T_{i,k-1} ~ h_{i-k+1} \;- \; h_i ~ T_{i-1, k-1}}{{h_{i-k+1} \; - \; h_i}} , ~ \;\;\;\;\;\;\; \; \; \; \; i=1, \ldots N\nonumber \\ \qquad \qquad \qquad \qquad \qquad \qquad k=1, \ldots i \end{aligned}$$

Here, $\Delta$ represents a user-defined integer value; Table 2 provides an example of the approximate value of the sum (48), with a specified $p_n^l \equiv p_5^4$ and $p_{n'}^l \equiv p_2^4$ and $t'-t = 1$ – once calculated directly, whereas the sum was aborted at a upper limit ($n_{\max }$) (column 1), the second time was extrapolated *via* an Aitken–Neville algorithm (column 5). For the extrapolation in column 5, the same upper summation limit was chosen such that columns 2 and 5 are directly comparable ( N $\Delta = n_{\max }$ ).

## Results

In this section, exemplary results are presented for the value of

52 $$\begin{aligned} \sum\kern -22.5pt\int \it{dx} ~ \frac{\rho _l(x)}{1-x} ~ p_{5}^l ~ \; p_{2}^l ~ e^{-iE(x)(t'-t)} \end{aligned}$$

for various *l* values (*l* = 0, 1, 4) and various values of $(t'-t)$. It is important to note that calculations with other values of *n*, *l* show similar results.

Our calculations were carried out using three procedures discussed in previous sections:

1. Gauss–Pollaczek quadrature along the real axis. The respective abscissae and weights result from diagonalization of the coefficient matrix with a tridiagonal structure as shown by (34). It is not required for the weight function $\rho _l (x)$ to be calculated explicitly, since it is implicitly part of the weight $w_i$; likewise, this incorporates the sum over the discrete spectrum into the Gauss quadrature, making its separate calculation obsolete. This results in 53
2. Gauss–Legendre quadrature along the real axis. For the calculation of the abscissae and weights, standard routines are used to calculate the roots of the Legendre polynomial with the Newton procedure.
3. Complex contour integration. In order to enable direct comparison with real axis integration, we chose the same Gauss–Legendre quadrature method.

Specifically, only 2 and 3 can be directly compared to each other, because the Gauss–Pollaczek quadrature approximates the complete term (29), including the sum over the discrete negative bound energies. Thus, for the calculation along the real axis and complex contour, the sum in (52) was calculated following the procedure described in the previous section and added to the integral over the continuum part in all Tables 3, 4 and 5.

For this calculation, a Java package (Apfloat) that allows calculations with any user-defined number of digits was used. Calculations were carried out with 40 digits to prevent rounding and truncation errors influencing the result. A particularly interesting aspect of these three integration procedures is the case $l = 0$. In this case, the convergence behavior should be least pronounced.

As a result, the following considerations can be given.

1. Convergence behavior improves in all three procedures with increasing *l* values (see Tables 3, 4, 5). This is to be expected due to the factor $1/k^{2l+2}$, and substantiates the qualitative statement from the previous section.
2. The convergence behavior of calculations with complex contour representations improves at higher values of $(t'-t)$; in the other two procedures, the opposite is valid.
3. If considering the value $l = 0$, the Gauss–Pollaczek procedure even converges poorly at $t'-t \sim 0$, although no oscillating behavior occurs and the integrand differs from the orthogonality relationship of the Pollaczek polynomials only by the factor $1 / (1-x)$. Even with 1200 abscissae, the result is accurate for only around three digits, i.e., an unexpected finding. At higher values of $t'-t$, the convergence behavior worsens—at this point, the oscillating character of the exponential function comes into play.
4. Gauss–Legendre quadrature along the real axis: Here, an improved convergence behavior occurs, with fewer abscissae than in the Gauss–Pollaczek quadrature. The reason for this probably lies in the distribution of the abscissae. In the Gauss–Pollaczek procedure, they have an accumulation point at $E = 0$, while they are more evenly distributed in the Gauss–Legendre procedure.
5. When considering the Gauss–Legendre quadrature of the complex contour integral, the number of abscissae is highest for $t'-t \sim 0$ to achieve a satisfactory result, whereas that for the value $t'-t \sim 0$ observed is only slightly worse than in the integration along the real axis.
6. In general, complex contour representation, combined with a Gauss–Legendre quadrature, yields the best result; however, the numerical input is high and the integrand must be evaluated using complex arguments.

## Conclusion and prospects

The evaluation of the Coulomb propagator on a L$^2$ basis can be accurately computed using extrapolation techniques for the bound state sum and mapping of the continuous part to a contour integral in the complex plane with subsequent Gauss quadrature. Direct comparison of integrations along the real axis and contour, requires that the same quadrature method is applied. Test cases give evidence that, for contour integration, the numerical method can be further improved by using a Gauss Hermite quadrature, thereby reducing the number of abscissae. Numerical efforts to evaluate contour integrals decrease with increasing time $t-t'$ and angular momentum *l*. Increasing $t-t'$ results in increasing damping, thus lowering the number of abscissae and the number of digits to achieve numerical accuracy. The same is true for increasing angular values *l*, there the integrand behaves like $1/k^{2l+2}$.

For practical applications, $N^2/2$ integrals were evaluated, where N is the maximum number of basis functions included in the expansion. Using the three-term recursion relation for the Pollaczek polynomials, the number of evaluations reduces to 2N.

The methods shown in this article can be used, for example, to propagate an electronic wavepacket released after a few cycles light pulse or in time-dependent perturbation theory. The computational effort is simplified when the initial state is a free state, i.e., it can be decomposed in a superposition of positive energy components, where the sum over bound states vanishes.

The L$^2$-Coulomb propagator can also be applied to draft a time-dependent approach of the J-Matrix scattering method. This leads to a L$^2$ analog of time-dependent R-Matrix theory, wherein the function space is split into an inner and an outer space. As such, time-dependent calculations on the femto- and attosecond photoionization range could be realized for one-electron systems.

## Supplementary information

Supplementary Information.
