# Supplementary material for: The quantum-mechanical Coulomb propagator in an L2 function representation
Source: Sci Rep. 2021 Sep 23;11:18997. doi: 10.1038/s41598-021-96925-0 (PMC8460653; doi:10.1038/s41598-021-96925-0)
Supplement: Supplementary file 1 — Supplementary Information. [file 41598_2021_96925_MOESM1_ESM.pdf]

Supplement: Calculation of the contour integral for $t' - t = 0$

The Pollaczek polynomials p_n^l and Pollaczek functions q_n^{l+} appearing in the L^2 expansion of the Coulomb propagator can be depicted as hypergeometric functions:[15]

$$p_n^l(\xi) \;=\; \frac{(n{+}2l{+}1)!}{(2l+1)!\;\; n!}\,(-\xi)^n\;\; {}_2F_1(-n,\; l{+}1{-}\frac{iz}{k};\; 2l+2;\; 1{-}\frac{1}{\xi^2}) \tag{A1}$$

$$q_n^{l+}(\xi) \;=\; -2\,\frac{\Gamma(l{+}1{-}\frac{iz}{k})}{\Gamma(n{+}l{+}2{-}\frac{iz}{k})}\,(-\xi)^{n+1}\,(n{+}2l{+}1)!\;\; {}_2F_1(-l{-}\frac{iz}{k},\; n{+}1;\; n{+}l{+}2{-}\frac{iz}{k};\; \xi^2) \tag{A2}$$

Due to the relationship $E = \frac{k^2}{2}$, the impulse is incorporated quadratically; thus, the inversion $k \to -k$ for positive values of E is also a solution of Schrödinger equation:

$$q_n^{l-}(\xi) \;=\; -2\,\frac{\Gamma(l{+}1{+}\frac{iz}{k})}{\Gamma(n{+}l{+}2{+}\frac{iz}{k})}\,(-\xi)^{-n-1}\,(n{+}2l{+}1)!\;\; {}_2F_1(-l{+}\frac{iz}{k},\; n{+}1;\; n{+}l{+}2{+}\frac{iz}{k};\; \frac{1}{\xi^2}) \tag{A3}$$

with

$$q_n^{l-}(\xi) \;=\; q_n^{l+}(\frac{1}{\xi}) \tag{A4}$$

Consequently, the product of the weight function ρ_l and the Pollaczek polynomial p_n^l can be expressed as linear combination of q_n^{l+} and q_n^{l-} :

$$\rho_l\; p_n^l \;=\; \frac{1}{2\pi i}\,(q_n^{l+} - q_n^{l-}) \tag{A5}$$

Through transformation onto the variable ξ with $x = -(\xi^2+1)/(2\xi)$ and $dx/d\xi = \frac{1}{2}(1/\xi^2 - 1)$, the integral over the Coulomb spectral density is as follows:

$$\int_{-1}^{+1} dx\; \frac{\rho_l(x)}{1-x}\; e^{-iE(x)(t'-t)}\; p_n^l\; p_{n'}^l \;=\; -\frac{1}{2\pi i}\oint d\xi\; \frac{1}{\xi}\;\frac{\xi-1}{\xi+1}\; e^{i\frac{\lambda^2}{8}\frac{(\xi-1)^2}{(\xi+1)^2}(t'-t)}\;\left[q_n^{l+}(\xi) - q_n^{l-}(\xi)\right]\; p_{n'}^l(\xi) \tag{A6}$$

The integration path encompasses the upper half of the unit circle in the complex ξ plane. When taking (A4) into consideration, the transformation onto $\xi' = 1/\xi$ results in:

$$\oint d\xi\; \frac{1}{\xi}\;\frac{\xi-1}{\xi+1}\; e^{i\frac{\lambda^2}{8}\frac{(\xi-1)^2}{(\xi+1)^2}(t'-t)}\; q_n^{l-}(\xi) \;=\; -\oint d\xi'\; \frac{1}{\xi'}\;\frac{\xi'-1}{\xi'+1}\; e^{i\frac{\lambda^2}{8}\frac{(\xi'-1)^2}{(\xi'+1)^2}(t'-t)}\; q_n^{l+}(\xi') \tag{A7}$$

As a result, the integral over the continuum part can be depicted as a closed contour integral over the unit circle in the complex ξ plane as follows:

$$\int_{-1}^{+1} dx\; \frac{\rho_e(x)}{1-x}\; e^{-iE(x)(t'-t)}\; p_n^l(x)\; p_{n'}^l(x) \;=\; -\frac{1}{2\pi i}\oint d\xi\; \frac{1}{\xi}\;\frac{\xi-1}{\xi+1}\; e^{i\frac{\lambda^2}{8}\frac{(\xi-1)^2}{(\xi+1)^2}(t'-t)}\; q_n^{l+}(\xi)\; p_{n'}^l(\xi) \tag{A8}$$

The advantage of of the closed contour representation in (A8) is, that the integral can be evaluated analytically for special cases by applying the residual theorem and considering the poles.

The function q_n^{l+} has poles at negative values of $E = -z^2/2n_b^2$, thus characterizing the Rydberg spectrum. These points lie within the unit circle on the real ξ axis; indeed, the Pollaczek polynomials p_n^l do not exhibit singularities and are thus continuous functions of ξ for $E > 0$. If expanding the product $q_n^{l+}\; p_{n'}^l$ under the assumption $n \geq n'$ at position $\xi = 0$ into a Taylor series, the following result is obtained:

$$q_n^{l+}\; p_{n'}^l|_{\xi=0} \;\;\sim\;\; (-\xi)^{n-n'+1}\left[1 + O(\xi^2)\right] \tag{A9}$$

The integrand (A8) has a pole at $\xi = -1$, due to the factor $1/(\xi+1)$, which also occurs in the argument of the exponential function.

For the case $t' - t = 0$, the integral (A8) can be evaluated analytically with the residual theorem and Cauchy principle value.

For this purpose, the integration path at the position $\xi = -1$ is deformed infinitesimally (see Fig. 2) such that the pole lies completely within its closed contour. Subsequently, crossing the limit of the function to the exact unit circle is conducted.

If setting $\xi = -1 + R\, e^{i\varphi}$ and expanding all terms of the integrand up to the first order in R, then crossing the limit of the function $R \to 0$, the result for (A8) is as follows:

$$q_n^{l+}(\xi = -1)\; p_{n'}^{l}(\xi = -1) \;+\; \sum \underset{\xi \to \xi(E=E_{n_b})}{Res} \left[\frac{1}{\xi}\, \frac{\xi - 1}{\xi + 1}\; q_n^{l+}(\xi)\; p_{n'}^{l}(\xi) \right] \tag{A10}$$

The Pollaczek polynomial $p_{n'}^{l}(-1)$ and the Pollaczek function $q_n^{l}(-1)$ can be analytically evaluated with the relationship that is valid for the hypergeometric function

$$_2F_1(a,b;c;0) = 1 \quad \text{and} \quad {}_2F_1(a,b;c;1) = \frac{\Gamma(c)\,\Gamma(c-a-b)}{\Gamma(c-a)\,\Gamma(c-b)} \tag{A11}$$

Overall, this results in:

$$\sum\!\!\!\!\!\!\int dx\; \frac{\rho_l(x)}{1-x}\; p_n^l\; p_{n'}^l \;=\; \frac{2(n_< + 2l + 1)}{(2l+1)\;\; n_<!} \tag{A12}$$

in which $n_<$ symbolizes the minimum (n, n').
